# Chemotherapeutics and Radiation Stimulate MHC Class I Expression through Elevated Interferon-beta Signaling in Breast Cancer Cells

**DOI:** 10.1371/journal.pone.0032542

**Published:** 2012-03-01

**Authors:** Shan Wan, Sidney Pestka, Ronald G. Jubin, Yi Lisa Lyu, Yu-Chen Tsai, Leroy F. Liu

**Affiliations:** 1 Department of Pharmacology, University of Medicine and Dentistry of New Jersey-Robert Wood Johnson Medical School, Piscataway, New Jersey, United States of America; 2 Department of Molecular Genetics, Microbiology and Immunology, University of Medicine and Dentistry of New Jersey-Robert Wood Johnson Medical School, Piscataway, New Jersey, United States of America; 3 PBL InterferonSource, Piscataway, New Jersey, United States of America; Mount Sinai School of Medicine, United States of America

## Abstract

Low doses of anticancer drugs have been shown to enhance antitumor immune response and increase the efficacy of immunotherapy. The molecular basis for such effects remains elusive, although selective depletion of T regulatory cells has been demonstrated. In the current studies, we demonstrate that topotecan (TPT), a topoisomerase I-targeting drug with a well-defined mechanism of action, stimulates major histocompatibility complex class I (MHC I) expression in breast cancer cells through elevated expression/secretion of interferon-β (IFN-β) and activation of type I IFN signaling. First, we show that TPT treatment elevates the expression of both total and cell-surface MHC I in breast cancer cells. Second, conditioned media from TPT-treated breast cancer ZR-75-1 cells induce elevated expression of cell-surface MHC I in drug-naïve recipient cells, suggesting the involvement of cytokines and/or other secreted molecules. Consistently, TPT-treated cells exhibit elevated expression of multiple cytokines such as IFN-β, TNF-α, IL-6 and IL-8. Third, either knocking down the type I interferon receptor subunit 1 (IFNAR1) or addition of neutralizing antibody against IFN-β results in reduced MHC I expression in TPT-treated cells. Together, these results suggest that TPT induces increased IFN-β autocrine/paracrine signaling through type I IFN receptor, resulting in the elevated MHC I expression in tumor cells. Studies have also demonstrated that other chemotherapeutic agents (e.g. etoposide, cisplatin, paclitaxel and vinblastine) similarly induce increased IFN-β secretion and elevated MHC I expression. In addition, conditioned media from γ-irradiated donor cells are shown to induce IFN-β-dependent MHC I expression in unirradiated recipient cells. In the aggregate, our results suggest that many cancer therapeutics induce elevated tumor antigen presentation through MHC I, which could represent a common mechanism for enhanced antitumor immune response through T cell cytotoxicity during metronomic chemotherapy, as well as increased efficacy of combined chemo- (or radio-)/immuno-therapy.

## Introduction

Cancer vaccines hold great promise for cancer therapy due to their highly specific, well tolerated, and long-lasting anti-tumor effect. However, it is well known that tumors develop various immune escape mechanisms to evade the host immune system, which could be the major reason accounting for the failure of immunotherapy [1].

Repeated low-dose chemotherapy (metronomic chemotherapy) has been shown to induce antitumor immune response [2]. Studies have also demonstrated that low doses of certain cancer chemotherapeutics and ionizing radiation can enhance the efficacy of immunotherapy (e.g. dendritic cell- and DNA vaccine-based immunotherapies) [3]. For example, pretreatment with low-dose cyclophosphamide (CTX), as a single agent or in combination with other anticancer drugs, has been shown to enhance cytotoxic T lymphocyte (CTL)-mediated antitumor immune response to tumor vaccination in animal models [4], [5]. In metastatic pancreatic cancer patients receiving a cell-based cancer vaccine, low-dose CTX administration has been shown to enhance antigen-specific CTL cytotoxicity [6]. It appears that chemotherapy could restore immune surveillance perhaps by breaking the tumor immune escape mechanisms. Indeed, low-dose chemotherapeutics have been observed to selectively deplete T regulatory cells that are known to represent a major tumor immune escape mechanism [7]–[10].

Another tumor immune escape mechanism involves blunted antigen presentation through reduced expression of major histocompatibility complex class I (MHC I) molecules on the tumor cell surface [11]. Consisting of a transmembrane α chain and β2 microglobulin (β2 m), the MHC I heterodimers present antigens to antigen-specific CTLs, resulting in cell killing [12]. While MHC I is present on the surfaces of all normal nucleated cells, deficiency of MHC I antigen presentation is very frequent in tumors [11]. Several studies have demonstrated low-dose chemotherapy can elevate antigen presentation by MHC I, which has been suggested to be a potential mechanism for immune sensitization of tumors [10], [13], [14].

Camptothecins (CPTs) such as topotecan (TPT) and irinotecan have been used in the clinic for the treatment of ovarian, colon, lung and other cancers (reviewed in [15]). The mechanism of tumor cell killing by CPTs has been well advanced. In the presence of CPTs, DNA topoisomerase I molecules are trapped on DNA as reversible Top1-CPT-DNA ternary complexes which arrest the advancing replication forks, resulting in S phase-specific killing of tumor cells (reviewed in [16], [17]). Because of its well characterized mechanism of tumor cell killing, we have employed TPT as a model to study tumor immune sensitization by cancer therapeutics. In the current studies, we have observed that TPT, as well as many other chemotherapeutics and ionizing radiation, induces elevated MHC I expression in breast cancer cells through induction of IFN-β, suggesting that elevated tumor antigen presentation through IFN-β autocrine/paracrine signaling could represent a common mechanism underlying tumor immune sensitization by cancer therapeutics.

## Results

### TPT elevates MHC I expression in breast cancer cells

**Figure 1:**
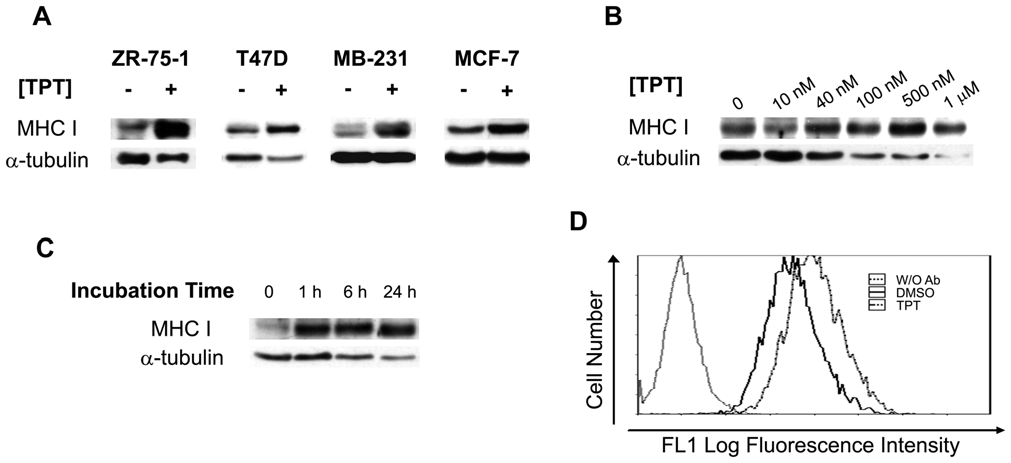
TPT induces elevated expression of both total and cell-surface MHC I in breast cancer cells. (**A**) TPT induces elevated expression of total cellular MHC I in breast cancer cells. Breast cancer cell lines ZR-75-1, T47D, MDA-MB-231 (MB-231) and MCF-7 were treated with TPT (40, 200, 20, and 40 nM, respectively) for 4 days. Expression of total cellular MHC I was measured by immunoblotting using a polyclonal antibody against MHC I. (**B**) The concentration effect of TPT on total cellular expression of MHC I. ZR-75-1 cells were treated with increasing concentrations of topotecan (10 nM to 1 µM) for 4 days, followed by immunoblotting for total cellular MHC I expression. (**C**) Acute (1 hr) TPT exposure stimulates total cellular expression of MHC I. ZR-75-1 cells were treated with TPT (40 nM) for increasing durations of time (0, 1, 6 and 24 hrs), followed by incubation in drug-free medium for a total combined incubation of 4 days. Total cellular expression of MHC I was measured by immunoblotting. (**D**) TPT induces elevated cell-surface expression of MHC I. ZR-75-1 cells were treated with 40 nM of TPT for 1 hr, followed by incubation in drug-free medium for 4 days. Cell-surface MHC I was measured by FACS analysis using the pan-MHC I monoclonal antibody, W6/32.

Previous studies have shown that MHC I expression was enhanced after treatment with cisplatin, fluorouracil, SN-38 or 5-aza-2′-deoxycytidine [13], [14], [18]. To test whether MHC I can also be induced by TPT, we treated breast cancer cells lines ZR-75-1, MCF-7, T47D and MDA-MB-231 with TPT for 4 days, followed by immunoblotting using an anti-MHC I polyclonal antibody. As shown in Fig. 1A, TPT induced elevated expression of total cellular MHC I in all four cell lines. We also performed a study with different concentrations of TPT in ZR-75-1 cells. As shown in Fig. 1B, at as low as 40 nM, TPT induced significant elevation of cellular MHC I expression. The decrease of α-tubulin levels in TPT-treated ZR-75-1 cells indicates cell loss/death due to the cytotoxic activity of TPT [19]. The fold-induction of MHC I upon normalization is about 4 at 40 nM of TPT (Fig. 1B). In a time-course study, ZR-75-1 cells were incubated with 40 nM of TPT for various times (0, 1, 6, and 24 hrs), followed by incubation in drug-free medium for a combined incubation time (drug treatment+drug-free incubation) of 4 days. As shown in Fig. 1C, MHC I expression was elevated (∼3-fold) after as short as 1 hr of TPT treatment. In addition to measuring total cellular MHC I, cell-surface expression of MHC I was also monitored by FACS using the pan-MHC I monoclonal antibody W6/32. As shown in Fig. 1D, 1 hr TPT treatment (40 nM) followed by 4-day incubation resulted in greatly elevated surface expression of MHC I (the mean fluorescence intensity increased from 7.16 to 13.1), suggesting elevated antigen presentation through MHC I in TPT-treated ZR-75-1 cells.

### TPT treatment of breast cancer cells increases IFN-β secretion

**Figure 2:**
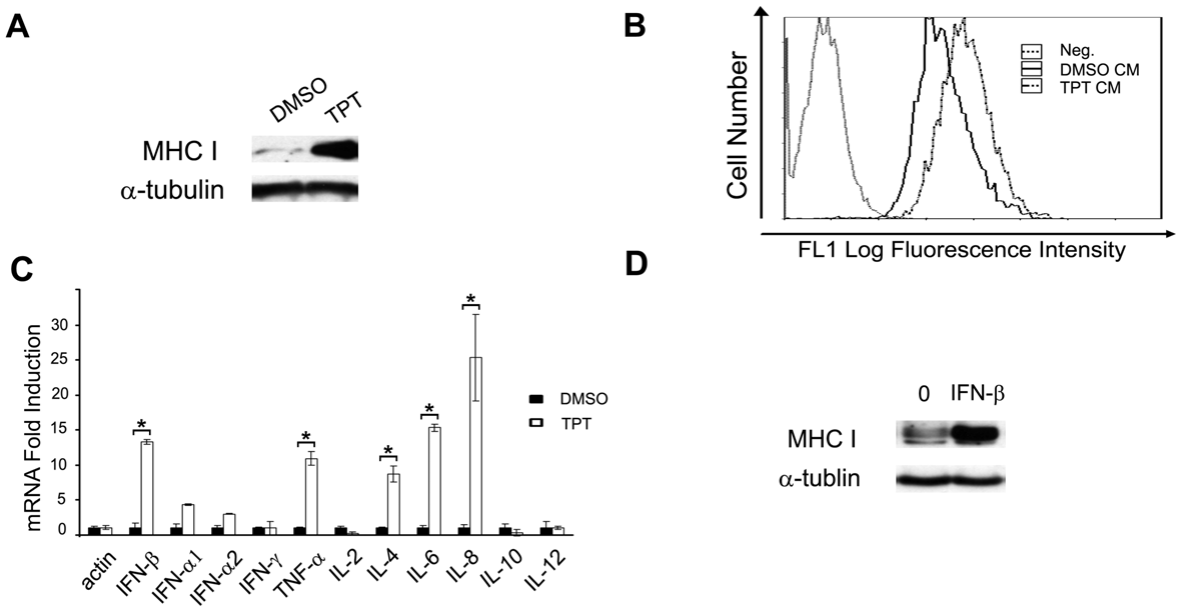
Increased secretion of IFN-β is responsible for TPT-induced MHC I expression. (**A**) Conditioned media from TPT-treated cells stimulate total cellular expression of MHC I in drug-naïve cells. ZR-75-1 cells were treated with 40 nM TPT for 1 hr, followed by incubation in drug-free medium for 4 days. Drug-naïve ZR-75-1 cells were then replenished with the conditioned media for another 2-day incubation. The total expression of cellular MHC I in recipient cells was measured by immunoblotting. (**B**) Conditioned media from TPT-treated cells stimulate cell-surface expression of MHC I in drug-naïve recipient cells. Recipient ZR-75-1 cells were treated with the conditioned media as described above, and the cell-surface MHC I expression was measured by FACS analysis. (**C**) TPT treatment increases mRNA levels of various cytokines in ZR-75-1 cells. Cells were treated with TPT (40 nM) for 1 hr, followed by incubation in drug-free medium for 3 days. Total RNAs were harvested for analysis by real-time RT-PCR. The experiments have been repeated twice. *: *p*-value<0.05. The error bar indicates standard deviation. (**D**) Purified IFN-β induces total cellular expression of MHC I. ZR-75-1 cells were treated with recombinant human IFN-β (500 U/ml) for 2 days. The total expression of cellular MHC I in recipient cells was measured by immunoblotting.

Tumor cells are known to secrete various cytokines, chemokines, as well as angiogenic and growth factors [20]. Chemotherapeutics such as doxorubicin and cisplatin have been shown to increase the secretion of cytokines such as IL-6 and IL-8, as well as other molecules from tumor cells [21]–[23]. To investigate a possible involvement of cytokines in the TPT-induced MHC I elevation, we harvested conditioned media from TPT-treated ZR-75-1 cells, and transferred them to drug-naïve ZR-75-1cells. As shown in Fig. 2, conditioned media from TPT-treated cells stimulated the expression of both total cellular (Fig. 2A, immunoblotting results) and cell-surface MHC I (Fig. 2B, FACS results) in drug-naïve recipient cells, indicating that soluble factors such as cytokines could be responsible for the MHC I induction.

Type I IFNs, including IFN-αs and IFN-β, are known strong regulators of MHC I expression [24], [25] and could be involved in TPT-induced MHC I expression. We measured the mRNA expression of IFN-α1, IFN-α2 and IFN-β, as well as some other cytokines in ZR-75-1 cells treated with TPT (40 nM for 1 hr followed by 3-day incubation in drug-free medium) using real-time RT-PCR. As shown in Fig. 2C and Table S1, the IFN-β level was elevated by 13-fold (Fig. 2C). Other cytokines, such as IFN-α1, IFN-α2, TNF-α, IL-1β, IL-4, IL-6, and IL-8, were also significantly induced by TPT treatment (*p*<0.05). Furthermore, secretion of IFN-β was confirmed and measured by an ELISA assay. As shown in Table 1, treatment (24 hrs followed by 3-day drug-free incubation) with TPT (40 nM) elevated IFN-β secretion by 4.0 fold.

**Table 1:** Cancer chemotherapeutics induce IFN-β secretion (ELISA assay).

	IFN-β (pg/ml)	Fold Increase
**DMSO**	25.0	1.0
**TPT**	99.8	4.0
**etoposide**	86.6	3.5
**cisplatin**	67.3	2.7
**paclitaxel**	68.4	2.7
**vinblastine**	71.1	2.8

ZR-75-1 cells were treated with topotecan (40 nM), etoposide (1 µM), cisplatin (6 µM), paclitaxel (3 µM), or vinblastine (6 nM) for 24 hrs, aspirated and washed, followed by incubation in drug-free medium for 3 days. Levels of IFN-β protein in the culture media were then measured by ELISA.

To verify the effect of IFN-β on MHC I expression, ZR-75-1 cells were treated with purified IFN-β (500 U/ml) and total protein expression of MHC I was measured by immunoblotting. As shown in Fig. 2D, purified IFN-β was shown to greatly increase MHC I expression.

### Activation of IFN-β signaling through the type I IFN receptor (IFNAR) is responsible for TPT-induced MHC I expression

**Figure 3:**
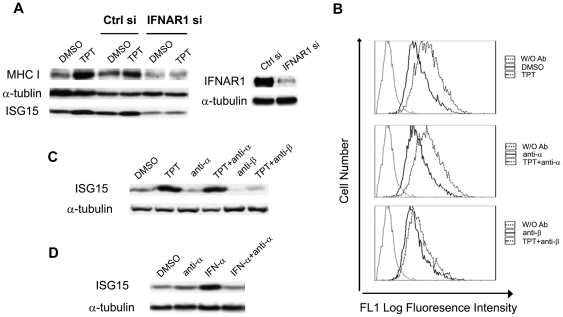
IFN-β signaling through type I IFN receptor is responsible for the TPT-induced MHC I expression. (**A**) IFNAR1 knockdown abolishes TPT-induced total cellular MHC I expression. On the left panel, ZR-75-1 cells were mock-transfected, or transfected with control siRNA or IFNAR1-specific siRNA. 48 hrs post-transfection, cells were treated with TPT (or 0.1% DMSO as control) for 1 hr, followed by incubation in drug-free medium for 4 days. Lysates were analyzed by immunoblotting. On the right panel, ZR-75-1 cells were transfected with control or IFNAR1-specific siRNA. 72 hrs post-transfection, cells were harvested and immunoblotted with an anti-IFNAR1 antibody. (**B**) Neutralizing antibody against IFN-β, but not IFN-α, blocks the TPT-induced cell-surface MHC I expression. ZR-75-1 cells were treated with TPT (40 nM or 0.1% DMSO as control) for 1 hr, followed by incubation in drug-free medium with or without neutralizing antibody against IFN-α (anti-α, 1.44×10^3^ NU/ml) or IFN-β (anti-β, 296 NU/ml). Cell-surface MHC I expression was measured by FACS analysis. (**C**) Neutralizing antibody against IFN-β, but not IFN-α, blocks the TPT-induced ISG15. Antibody and TPT treatments of ZR-75-1 cells were performed exactly the same as described in (**B**). Lysates were immunoblotted for ISG15 expression. (**D**) Neutralizing antibody against IFN-α inhibits IFN-α-induced ISG15 expression. ZR-75-1 cells were co-incubated with IFN-α (100 U/ml) in the presence or absence of anti-IFN-α antibody (1.44×10^3^ NU/ml) for 2 days. Cells were then harvested and immunoblotted with an anti-ISG15 antibody.

To determine if IFN-β signaling is involved in the TPT-induced MHC I expression, interferon (alpha, beta and omega) receptor (subunit) 1 (IFNAR1) was silenced by IFNAR1-specific siRNA in ZR-75-1 cells. As shown in Fig. 3A, IFNAR1 knockdown in ZR-75-1 cells abolished the TPT-induced MHC I expression, suggesting that autocrine/paracrine signaling through IFNAR plays an important role in the MHC I induction by TPT. The knockdown efficiency (about 80%) of IFNAR1 was determined by immunoblotting (see Fig. 3A, right panel).

The role of secreted IFN-β in MHC I expression was also assessed by using the neutralizing antibody against IFN-β in TPT-treated ZR-75-1 cells. As shown in Fig. 3B, after 1 hr treatment with TPT, ZR-75-1 cells were incubated in drug-free medium in the presence of the neutralizing antibody against IFN-α or IFN-β for 4 days. Only the neutralizing antibody against IFN-β, but not IFN-α, blocked the TPT-induced cell-surface MHC I expression (Fig. 3B, FACS analysis), suggesting that elevated secretion of IFN-β from drug-treated cells is responsible for MHC I upregulation. Also, the neutralizing antibody against IFN-β, but not IFN-α, was shown to block ISG15 (an IFN-inducible gene) [26] induction in TPT-treated ZR-75-1 cells (Fig. 3C), suggesting that IFN signaling is primarily activated by IFN-β, but not IFN-α, in TPT-treated ZR-75-1 cells. The anti-IFN-α antibody was shown to be functional as it blocked IFN-α-induced expression of ISG15 (Fig. 3D).

In the aggregate, these results suggest that the induction of IFN-β autocrine signaling by TPT treatment is primarily responsible for the TPT-induced MHC I expression.

### TPT-induced MHC I expression requires active DNA synthesis

**Figure 4:**
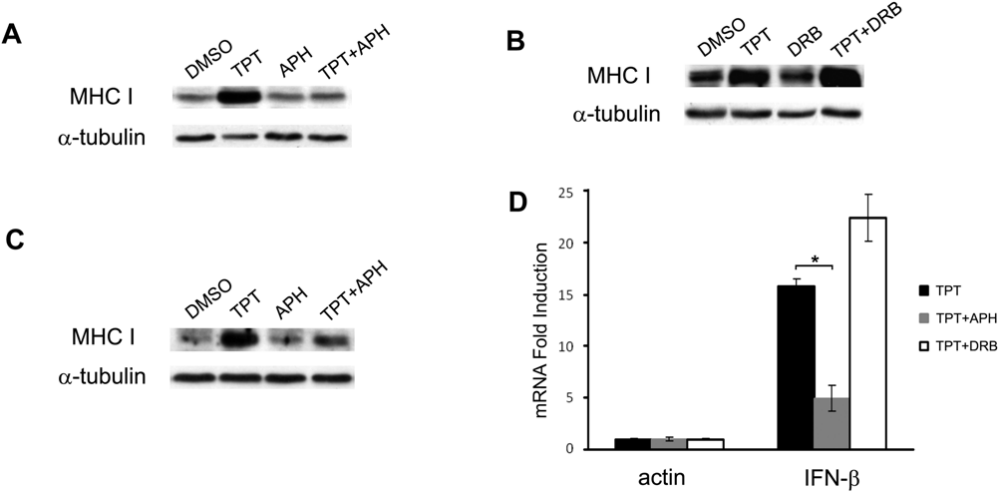
TPT-induced elevation of MHC I and IFN-β is DNA replication-, but not transcription-, dependent. (**A**) TPT-induced MHC I expression require active DNA synthesis. ZR-75-1 cells were pre-treated with APH (10 µM) for 30 min, followed by 0.1% DMSO or TPT (40 nM) treatment for 1 hr, and subsequent incubation in drug-free medium for 4 days. Expression of MHC I was then measured by immunoblotting. (**B**) TPT-induced MHC I expression is independent of transcription. ZR-75-1 cells were pre-treated with the transcription inhibitor DRB (150 µM) for 30 min, and then treated with TPT as described in (**A**). Expression of MHC I was measured by immunoblotting. (**C**) Conditioned medium-induced MHC I expression in recipient cells requires active DNA synthesis in TPT-treated donor cells. Recipient ZR-75-1 cells were incubated with conditioned media from TPT-treated donor cells as described in (**A**). The total expression of cellular MHC I in recipient cells was then measured by immunoblotting. (**D**) TPT-induced IFN-β mRNA expression requires active DNA synthesis. ZR-75-1 cells were pretreated with APH (10 µM) or DRB (150 µM) for 30 min prior to co-incubation with TPT (40 nM, 1 hr), followed by drug-free incubation for 3 days. Total RNAs were isolated for real-time RT-PCR analysis. The experiments have been repeated three times. *: *p*-value<0.05. The error bar indicates standard deviation.

The major cellular effects of CPTs including DNA damage response and cell death have been attributed to the arrest of DNA replication forks by drug-stabilized topoisomerase I-DNA covalent adducts [27]. To test whether TPT-induced MHC I expression is a downstream event of drug-induced replication fork arrest, cells were treated with TPT in the presence of either the replication inhibitor aphidicolin (APH) or the transcription inhibitor 5,6-dichloro-1-β-D-ribobenzimidazole (DRB) for 30 min, followed by further incubation in drug-free medium for 4 days. As shown in Fig. 4A and 4B, the presence of APH, but not DRB, abolished TPT-induced MHC I expression in ZR-75-1. Co-treatment of TPT with APH in the donor cells was also shown to abolish the conditioned media-induced MHC I expression in the drug-naïve recipient cells (Fig. 4C). Furthermore, the effect of APH and DRB on IFN-β mRNA expression in TPT-treated ZR-75-1 cells was determined using real-time RT-PCR. As shown in Fig. 4D, APH, but not DRB, significantly blocked the TPT-induced IFN-β mRNA expression (*p*<0.05). Together, these results suggest that IFN-β secretion and subsequent MHC I elevation in TPT-treated cells require active DNA synthesis, implicating the formation of Top1 cleavage complexes and their arrest of DNA replication forks being responsible for these TPT effects.

### TPT-induced MHC I expression depends on NF-κB activation, but not caspase activation

**Figure 5:**
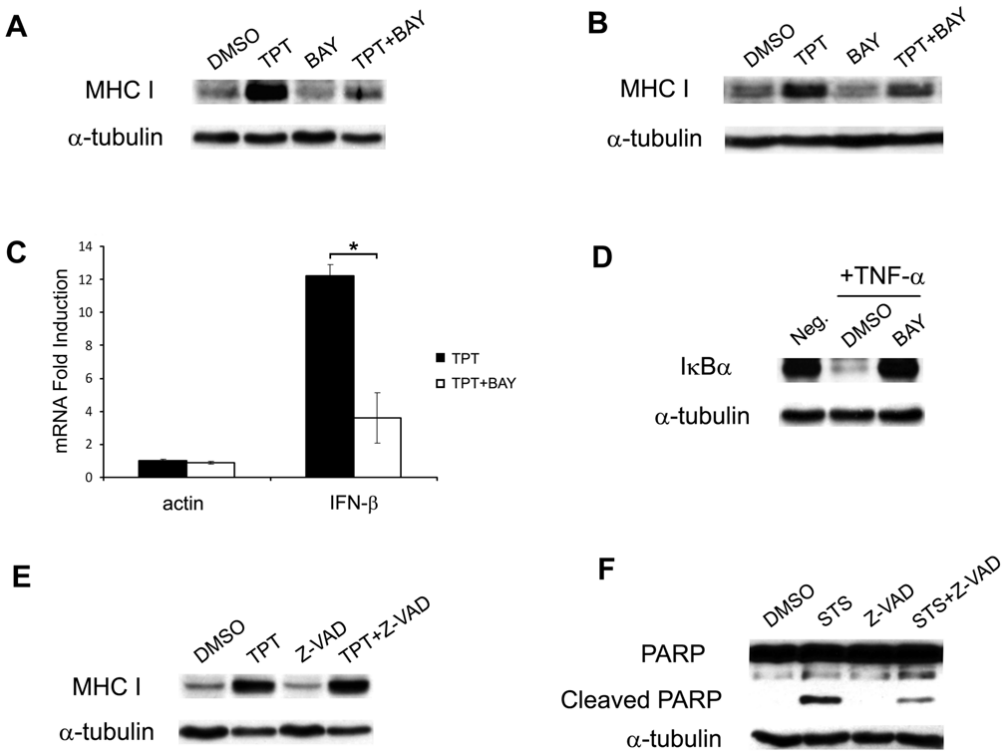
TPT-induced MHC I expression requires NF-κB activation, but not apoptotic caspases. (**A**) The NF-κB inhibitor BAY 11-7085 (BAY) blocks TPT-induced MHC I expression. ZR-75-1 cells were pretreated with BAY (10 µM) for 30 min, followed by co-incubation with 0.1% DMSO or TPT (40 nM) for 1 hr, and subsequent drug-free incubation for 4 days. Total cellular MHC I was then analyzed by immunoblotting. (**B**) The conditioned medium-induced MHC I in recipient cells requires NF-κB activation in TPT-treated donor cells. Drug- naïve recipient ZR-75-1 cells were incubated with the conditioned medium from TPT-treated donor cells as described in (**A**). Expression of total cellular MHC I in recipient cells was then analyzed by immunoblotting. (**C**) TPT-induced IFN-β mRNA expression requires NF-κB activation. ZR-75-1 cells were pretreated with BAY (10 µM) for 30 min prior to co-incubation with TPT (40 nM, 1 hr), followed by drug-free incubation for 3 days. Total RNAs were isolated for real-time RT-PCR analysis. The experiments have been repeated three times. *: *p*-value <0.05. The error bar indicates standard deviation. (**D**) BAY specifically blocks IκBα degradation induced by TNF-α treatment. ZR-75-1 cells were treated with TNF-α (10 ng/ml) for 10 min, in the presence or absence of BAY (10 µM). Expression of IκBα was measured by immunoblotting. (**E**) TPT-induced MHC I expression is independent of caspase activation. ZR-75-1 cells were pretreated with the pan-caspase inhibitor Z-VAD-FMK (Z-VAD, 10 µM) for 1 hr prior to co-incubation with TPT (40 nM, 1 hr), followed by continued incubation in drug-free medium for 4 days. Cell lysates were then immunoblotted with the anti-MHC I antibody. (**F**) Staurosporine-induced PAPR-1 cleavage requires caspase activation. ZR-75-1 cells were pretreated with Z-VAD-FMK (10 µM) for 1 hr followed by co-incubation with staurosporine (STS, 0.5 µM) for 6 hrs. PARP-1 cleavage was then measured by immunoblotting.

NF-κB regulates expression of many cytokines, such as IFN-β, TNF-α, IL-1β, IL-6 and IL-8, by activating their gene transcription through direct binding to the promoters [28], [29]. CPTs as well as many cancer therapeutics are known to activate NF-κB [30]–[33]. It has been demonstrated that NF-κB activation, like DNA damage and cell death, is induced by CPTs in S-phase and is dependent on the arrest of replication forks by CPT-induced topoisomerase I-DNA covalent adducts [30], [34]. To investigate whether the TPT-induced expression of IFN-β and MHC I is due to NF-κB activation, a NF-κB inhibitor, BAY 11-7085 [35], was employed. As shown in Fig. 5A, BAY 11-7085 blocked the TPT-induced MHC I expression in ZR-75-1 cells, suggesting a key role of NF-κB in TPT-induced MHC I expression. Furthermore, conditioned media from ZR-75-1 cells co-treated with BAY 11-7085 and TPT were unable to induce MHC I expression when transferred to the drug-naïve recipient cells, suggesting that NF-κB activation in the drug-treated donor cells is responsible for MHC I expression in the recipient cells (Fig. 5B). This result points to a role of activated NF-κB in elevating IFN-β expression/secretion in the donor cells, which is responsible for the induced MHC I expression in the recipient cells. Indeed, BAY 11-7085 co-treatment significantly abolished IFN-β mRNA induction as measured by real-time RT-PCR (*p*<0.05) (Fig. 5C). As a control, we demonstrated the levels of BAY 11-7085 used in this study specifically blocks IκBα degradation induced by direct TNF-α treatment in ZR-75-1 cells (Fig. 5D).

CPTs are known to induce apoptosis in addition to mitotic catastrophe [36], [37]. In Fig. 5E, we show that the general caspase inhibitor Z-VAD-FMK did not block the TPT-induced MHC I expression, suggesting that the MHC I induction is independent of the apoptotic pathway. As a control, Z-VAD-FMK was shown to block staurosporine-induced PARP-1 cleavage (Fig. 5F).

Taken together, these results suggest that the TPT-induced IFN-β secretion and MHC I expression is dependent on NF-κB activation, but independent of caspase activation.

### Cancer chemotherapeutics and ionizing radiation induce MHC I expression and IFN-β secretion

**Figure 6:**
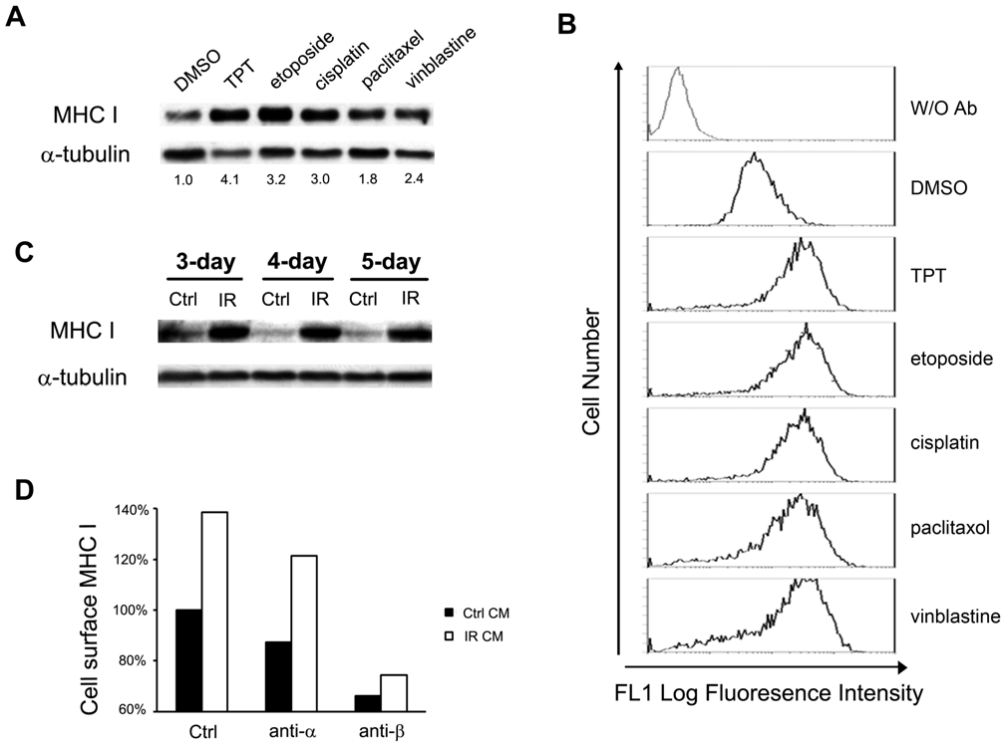
Cancer chemotherapeutics and ionizing radiation induce MHC I expression in breast cancer cells. (**A**) Cancer chemotherapeutics induce elevated expression of total cellular MHC I in breast cancer ZR-75-1 cells. Cells were treated with topotecan (40 nM), etoposide (1 µM), cisplatin (6 µM), paclitaxel (3 µM), and vinblastine (6 nM) for 24 hrs, followed by incubation in drug-free medium for 3 days. Expression of total cellular MHC I was measured by immunoblotting. (**B**) Cancer chemotherapeutics induce cell-surface expression of MHC I in ZR-75-1 cells. ZR-75-1 cells were treated with different anticancer drugs as described in (**A**). Expression of cell-surface MHC I was then determined by FACS analysis. (**C**) Conditioned media from γ-irradiated cells induce elevated total cellular expression of MHC I in radiation-naïve recipient cells. Donor (ZR-75-1) cells were irradiated with γ-ray (2 Gy) for 3, 4, and 5 times. The conditioned media were then transferred to radiation-naïve recipient (ZR-75-1) cells. After 48 hrs, cell lysates were harvested for immunoblotting analysis. (**D**) Induction of MHC I expression in drug naïve recipient cells depends on IFN-β in the conditioned medium from irradiated donor cells. Radiation-naïve recipient (ZR-75-1) cells were incubated with the conditioned medium from irradiated (2 Gy×4) donor (ZR-75-1) cells in the presence of the neutralizing antibody against IFN-α (anti-α) or IFN-β (anti-β) for two days. Cell-surface MHC I in the recipient (ZR-75-1) cells was then measured by FACS analysis.

To test whether elevated MHC I expression through IFN-β secretion/signaling is a general phenomenon induced by cancer chemotherapeutics, ZR-75-1 cells were treated with etoposide (a topoisomerase II inhibitor), cisplatin (a DNA damaging agent), paclitaxel (a microtubule stabilizer) and vinblastine (a microtubule destabilizer). As shown in Fig. 6A, all of these drugs, like TPT, induced elevated expression of total cellular MHC I as evidenced by immunoblotting. Furthermore, the cell-surface expression of MHC I was also found to be greatly enhanced by these cancer chemotherapeutics (Fig. 6B). Moreover, we measured the protein levels of IFN-β in the media of drug-treated ZR-75-1 cells using ELISA assay. As shown in Table 1, treatments (24 hrs followed by 3-day drug-free incubation) with etoposide (1 µM), cisplatin (6 µM), paclitaxel (3 µM) and vinblastine (6 nM) elevated IFN-β secretion by 3.5, 2.7, 2.7 and 2.8 folds, respectively. These results suggest that tumor cell MHC I expression and IFN-β secretion may be commonly induced by many different types of cancer chemotherapeutic agents.

Since ionizing radiation, like TPT, is known to induce DNA double-strand breaks and NF-κB activation, we investigated whether ionizing radiation can similarly induce IFN-β expression/secretion and elevate MHC I expression. We showed that conditioned media from irradiated ZR-75-1 cells, when transferred to unirradiated recipient cells, increased the expression of both total cellular (Fig. 6C, immunoblotting results) and cell-surface (Fig. 6D, FACS results) MHC I in the recipient cells, suggesting that MHC I elevation in the recipient cells is due to secreted soluble factors such as cytokines from the irradiated donor cells. Indeed, when neutralizing antibody against IFN-β, but not IFN-α, was added to the conditioned media from irradiated cells, much reduced expression of MHC I was observed in the unirradiated recipient cells (Fig. 6D). Collectively, these results suggest that IFN-β-mediated MHC I expression may be commonly induced by both cancer chemotherapeutics and ionizing radiation.

## Discussion

Our results demonstrate that the topoisomerase I-targeting anticancer drug topotecan (TPT) induces elevated secretion of multiple cytokines and increased MHC I expression in breast cancer cells. Silencing of IFN-β by siRNA or addition of purified IFN-β (see Fig. 2D) had a large effect on MHC class I expression, suggesting a causal relationship between elevated IFN-β signaling and increased MHC I expression in TPT-treated breast cancer cells. By contrast, silencing of the TNF-α receptor by siRNA did not affect MHC class I expression in TPT-treated ZR-75-1 cells, and addition of purified TNF-α (or IL-6) to ZR-75-1 cells had no observable effect on MHC I expression (slight increase in MHC class I expression was observed only at a high concentration (i.e. 5 ng/ml) of TNF-α (Shan Wan and Leroy F. Liu; unpublished results). Studies using the conditioned media from TPT-treated tumor cells further support the notion that increased secretion of IFN-β can elevate MHC I expression through autocrine/paracrine signaling.

Our studies have also demonstrated that TPT-induced IFN-β secretion and MHC I expression require active DNA synthesis since co-treatment (1 hr, followed by further incubation in drug-free medium for 4 days) with the replication inhibitor aphidicolin, but not the transcription inhibitor DRB, specifically abrogates this stimulatory effect. It has been well documented that tumor cell killing by CPTs occurs specifically in S phase and requires active DNA synthesis, as co-treatment with the replication inhibitors protects tumor cells from CPT cytotoxicity [19]. In addition, CPTs are known to activate NF-κB in S phase, which also requires active DNA synthesis [30], [34]. It has been proposed that CPT-induced Top1-DNA covalent adducts arrest the advancing replication forks, resulting in tumor cell death as well as DNA damage responses including activation of NF-κB [16], [30]. The transcription factor NF-κB is a major stress sensor that responds to various stimuli [28], [38]. Activation of NF-κB has been shown to induce diverse target genes at the transcription level, including multiple cytokines such as TNF-α, IL-1β, IFN-β, IL-6, IL-8 and others [28], [29]. It is noteworthy that only the promoter of IFN-β, but IFN-α, contains a NF-κB binding site [29]. Our inhibitor studies have suggested that TPT-induced MHC I expression is dependent on NF-κB activation. It seems likely that TPT-induced MHC I expression is due to the activation of the NF-κB/IFN-β/MHC I signaling axis. In this regard, it is interesting to point out that many cancer chemotherapeutics (e.g. etoposide, cisplatin, paclitaxel and vinblastine) and ionizing radiation are known to activate NF-κB [31]–[33], [39]. Consequently, elevated MHC I expression induced by other cancer therapeutics as demonstrated in our studies could also be due to the activation of the same NF-κB/IFN-β/MHC I signaling axis.

Selective depletion of T regulatory cells by certain cancer therapeutics has been proposed to be a key mechanism for enhanced antitumor immune response [40]. However, increasing evidence has also pointed to activation of type I IFN signaling as another mechanism for chemotherapy-enhanced antitumor immunity in mice [5], [41]–[43]. For example, cyclophosphamide administration has been shown to stimulate antigen-specific CTL response in mice vaccinated with a peptide antigen [5]. However, in IFNAR knockout mice, cyclophosphamide-induced stimulation of antigen-specific T cells was diminished, suggesting a critical role of type I IFN signaling in the immune-modulation by anticancer drugs [5]. Most significantly, the efficacy of adoptive immunotherapy is greatly reduced in mice co-treated with anti-IFN-α/β antibody [43]. Proposed explanations for the involvement of type I IFN signaling in chemotherapy-induced antitumor immunity include augmentation of memory T cells [41], increased tumor infiltrating T lymphocytes [42], and enhanced clonal expansion of antigen-specific CTLs [5]. Our results suggest that elevated tumor cell antigen presentation through MHC I may represent yet another mechanism for chemotherapy-enhanced antitumor immunity through increased IFN-β autocrine/paracrine signaling. It is possible that our observation is also relevant to the enhanced antitumor immune response during metronomic chemotherapy (repeated low-dose administration of anticancer drugs) [2].

Tumor irradiation has been reported to enhance the antitumor efficacy of immunotherapy in mice [44], [45]. However, the molecular basis for the radio-immune sensitization remains unclear. Our current studies have demonstrated that ionizing radiation, like cancer chemotherapeutics, induces elevated expression of MHC I in an IFN-β-dependent manner in breast cancer cells *in vitro*. It seems likely that ionizing radiation may also activate the same NF-κB/IFN-β/MHC I signaling axis, resulting in elevated tumor antigen presentation through MHC I and hence increased tumor cell killing by CTLs, which could represent a potential mechanism for radio-immune sensitization.

Our studies have demonstrated that many cytokines, in addition to IFN-β, are induced by TPT-treated tumor cells *in vitro*. It seems possible that elevated secretion of these cytokines by tumor cells, in addition to stromal cells, may also contribute to the tumor microenvironment in patients receiving chemo- or radio-therapy. These cytokines could exert diverse effects on local and distal tumor cells. For example, a “chemotherapy-induced bystander” phenomenon has been described [46]: conditioned media from chloroethylnitrosourea (CENU)-treated tumor cells have been shown to inhibit the growth of untreated tumor cells, suggesting the presence of growth-inhibitory cytokines/secreted molecules in the conditioned media. Further, metronomic administration of various chemotherapeutic agents, such as cyclophosphamide, methotrexate, paclitaxel, and vinblastine, has also been found to inhibit angiogenesis, partially contributed by secreted TSP1 (as reviewed in [47]). Additionally, the cytokine paracrine/autocine signaling could contribute to the well-known radiation-induced bystander effects, in which unirradiated tumor cells exhibit biological effects of irradiation (e.g. cell death and DNA damage response) due to signaling from nearby irradiated tumors [48]. Moreover, tumor irradiation has been shown to promote angiogenesis, and local and distal metastasis (as reviewed in [49]). Clearly, further studies are needed to better understand the roles of tumor-secreted cytokines in immune modulation and various bystander effects during chemo- or radio-therapy.

## Materials and Methods

### Cells and cell culture

Breast cancer cell lines ZR-75-1 and T47D were cultured in complete RPMI, while MDA-MB-231 and MCF-7 in complete DMEM. Both media (Sigma-Aldrich) were supplemented with 10% Fetalplex (Gemini Bio-Products), L-glutamine (2 mM), penicillin (100 units/ml) and streptomycin (100 µg/ml). Cells were maintained in a 37°C incubator with 5% CO_2_. All the cell lines were obtained from American Type Culture Collection, and were authenticated by the supplier.

### Chemicals and reagents

All anticancer drugs (i.e. topotecan, etoposide, cisplatin, paclitaxel, and vinblastine), the replication inhibitor aphidicolin and the transcription inhibitor 5, 6-dichloro-1-b-D-ribobenzimidazole, and apoptosis inducer staurosporine were purchased from Sigma-Aldrich, and stored in DMSO (except for cisplatin which is stored in water containing 0.9% NaCl). The pan-caspase inhibitor Z-VAD-FMK was purchased from Promega. The NF-kB inhibitor BAY 11-7085 was purchased from BIOMOL International. Purified recombinant proteins IFN-α and IFN-β, and neutralizing antibodies against IFN-α and IFN-β, were from PBL InterferonSource.

### Ionizing radiation

1.2×10^7^ breast cancer ZR-75-1 cells were seeded in 150 mm culture dishes 24 hrs prior to irradiation. Cells (80–90% confluent) were irradiated with 2 Gy gamma ray every 24 hrs for 3, 4 and 5 times, and were replenished with fresh media (15 ml) 1 hr after the last irradiation. The (conditioned) media were then collected 24 hrs later. Collected (conditioned) media were passed through 0.45 µm syringe filters to remove dead cells and debris, followed by storage in a −20°C freezer.

### Immunoblotting

Immunoblotting was performed as described previously [50]. Briefly, cells were lysed with 6× Laemmli SDS gel sample buffer, followed by boiling for 10 min. Cellular proteins were fractionated by SDS-PAGE, transferred to nitrocellulose membrane (Whatman), and probed with antibodies against MHC Class I (rabbit polyclonal anti-MHC I heavy chain antibody; Santa Cruz), α-tubulin (Developmental Studies Hybridoma Bank), IκB-α (Cell Signaling), IFNAR1 (Abcam), PAPR (Cell signaling), or ISG15 (raised against human ISG15). Proteins were visualized using SuperSignal West Pico Chemiluminescent Substrate (Pierce).

### Secreted IFN- β Measurement

ZR-75-1 cells were treated with topotecan, etoposide, cisplatin, paclitaxel, and vinblastine for 24 hrs, followed by incubation in drug-free medium for 3 days. Levels of IFN-β protein in the culture media were then measured using a *VeriKine*-*HS*™ Human Interferon-Beta Serum ELISA kit (PBL InterferonSource) according to the manufacturer's protocol.

### Real-time RT-PCR

Total RNAs were isolated with Trizol reagent (Invitrogen) and the RNeasy Mini Kit (Qiagen). About 1–2 µg of total RNAs were used for the synthesis of first-strand cDNA using the SuperScript III First-Strand Synthesis System (Invitrogen). Real-time RT-PCR was performed in the 7900HT Fast Real-Time PCR System (Applied Biosystems) with the use of SYBR Green PCR Master Mix (Applied Biosystems). Results were analyzed with SDS 2.2 software using the ^−Δ*CT*^ method [51]. The following primers were used: β-actin-F (5′- GGCACCCAGCACAATGAAGATCAA-3′) and β-actin-R (5′- ACTCGTCATACTCCTGCTTGCTGA-3′); IFN-α_1_-F (5′- AGGAGGAGTTTGATGGCAACCAGT-3′) and IFN-α_1_-R (5′- TGCTGGTAGAGTTCGGTGCAGAAT-3); IFN-α_2_-F (5′- AAGGACTCATCTGCTGCTTGGGAT-3′) and IFN-α_2_-R (5′- TCACACAGGCTTCCAGGTCATTCA-3′); IFN-β-F (5′- TGTGGCAATTGAATGGGAGGCTTG-3′) and IFN-β-R (5′- TCTCATAGATGGTCAATGCGGCGT-3′); IFN-γ-F (5′- TGCAGGTCATTCAGATGTAGCGGA-3′) and IFN-γ-R (5′- TGTCTTCCTTGATGGTCTCCACACTC-3′); IL-1β-F (5′- TCTGTACCTGTCCTGCGTGTTGAA-3′) and IL-1β-R (5′- TGCTTGAGAGGTGCTGATGTACCA-3′); IL-2-F (5′- ATCCCAAACTCACCAGGATGCTCA-3′) and IL-2-R (5′- GCACTTCCTCCAGAGGTTTGAGTTCT-3′); IL-4-F (5′- TACAGCCACCATGAGAAGGACACT-3′) and IL-4-R (5′- ACGTACTCTGGTTGGCTTCCTTCA-3′); IL-6-F (5′- TCAATGAGGAGACTTGCCTGGTGA-3′) and IL-6-R (5′- TACTCATCTGCACAGCTCTGGCTT-3′); IL-10-F (5′- AAGCTGAGAACCAAGACCCAGACA-3′) and IL-10-R (5′- AAAGGCATTCTTCACCTGCTCCAC-3′); IL-12-F (5′- TGCAGGCCCTGAATTTCAACAGTG-3′) and IL-12-R (5′- GTCACTGCCCGAATTCTGAAAGCA-3′); TNF-α-F (5′- AAGCCCTGGTATGAGCCCATCTAT-3′) and TNF-α-R (5′- ATGATCCCAAAGTAGACCTGCCCA-3′).

### siRNA knockdown

Cells were transfected with control siRNA(SIC001, Sigma-Aldrich) or IFNAR1 siRNA(s783, Ambion) with Oligofectamine transfection reagent (Invitrogen), as described previously [50].

### FACS analysis

Expression of cell-surface MHC I was measured using pan-MHC Class I reactive monoclonal antibody W6/32 (Abcam) and Cy2-conjugated goat anti-mouse IgG secondary antibody (Jackson Immunoresearch Laboratories). Briefly, cells were trypsinized, washed with ice-cold PBS containing 1% sodium azide (Sigma-Aldrich), and incubated with primary antibody for 30 min on ice, followed by 30 min incubation with the secondary antibody. After washing, cells were analyzed by a flow cytometer (Beckman Coulter FC500 Analyzer).

## Supporting Information

Table S1 TPT treatment increases expression levels of cytokine mRNAs in ZR-75-1 cells. ZR-75-1 cells were treated with TPT (40 nM) for 1 hr, followed by incubation in drug-free medium for 3 days. Total RNAs were isolated for real-time RT-PCR analysis. Ct values for each gene are recorded in the table, and −ΔΔCt was calculated according to [51]. The results are representative of three independent experiments.
(DOCX)
