# Supplementary material for: Chemotherapeutics and Radiation Stimulate MHC Class I Expression through Elevated Interferon-beta Signaling in Breast Cancer Cells
Source: PLoS One. 2012 Mar 1;7(3):e32542. doi: 10.1371/journal.pone.0032542 (PMC3291570; doi:10.1371/journal.pone.0032542)
Supplement: Table S1 — TPT treatment increases expression levels of cytokine mRNAs in ZR-75-1 cells. ZR-75-1 cells were treated with TPT (40 nM) for 1 hr, followed by incubation in drug-free medium for 3 days. Total RNAs were isolated for real-time RT-PCR analysis. Ct values for each gene are recorded in the table, and −ΔΔCt was calculated according to [51]. The results are representative of three independent experiments. (DOCX) [file pone.0032542.s001.docx]

**Supplementary Table S1**

|  | **β-actin** | **IFN-β** | **IFN-α1** | **IFN-α2** | **IFN-γ** | **TNF-α** | **IL-1β** | **IL-2** | **IL-4** | **IL-6** | **IL-8** | **IL-10** | **IL-12** |
| --- | --- | --- | --- | --- | --- | --- | --- | --- | --- | --- | --- | --- | --- |
| **Ct/DMSO** | 22.4 | 31.1 | 36.0 | 33.6 | 39.7 | 34.8 | >50 | 43.6 | 44.4 | 39.4 | 31.2 | 39.5 | 32.2 |
| **Ct/TPT** | 23.6 | 28.6 | 35.1 | 33.3 | 43.3 | 32.6 | 39.7 | 46.8 | 42.7 | 36.7 | 27.7 | 48.1 | 33.4 |
| **−ΔΔCt** | 0 | 3.7 | 2.2 | 1.5 | -2.4 | 3.4 | >11.6 | -2.0 | 3.0 | 3.9 | 4.7 | -7.4 | -0.1 |

TPT treatment increases expression levels of cytokine mRNAs in ZR-75-1 cells. ZR-75-1 cells were treated with TPT (40 nM) for 1 hr, followed by incubation in drug-free medium for 3 days. Total RNAs were isolated for real-time RT-PCR analysis. Ct values for each gene are recorded in the table, and –ΔΔCt was calculated according to [[51](#_ENREF_51)]. The results are representative of three independent experiments.
